# Nonhormonal therapy for endometriosis based on energy metabolism regulation

**DOI:** 10.1530/RAF-21-0053

**Published:** 2021-11-25

**Authors:** Hiroshi Kobayashi, Hiroshi Shigetomi, Shogo Imanaka

**Affiliations:** 1 Department of Obstetrics and Gynecology, Nara Medical University, Kashihara, Japan; 2 Department of Gynecology, Ms.Clinic MayOne, Kashihara, Japan; 3 Aska Ladies Clinic, Nara, Japan

**Keywords:** endometriosis, glycolysis, hypoxia, metabolism, oxidative phosphorylation, Warburg effect

## Abstract

Ovarian function suppression is the current pharmacotherapy of endometriosis with limited benefit and adverse effects. New therapeutic strategies other than hormonal therapy are developed based on the molecular mechanisms involved in the hypoxic and oxidative stress environments and metabolism unique to endometriosis. A literature search was performed between January 2000 and March 2021 in the PubMed database using a combination of specific terms. Endometriosis-associated metabolic changes have been organized into four hallmarks: (1) glucose uptake, (2) aerobic glycolysis, (3) lactate production and accumulation, and (4) metabolic conversion from mitochondrial oxidative phosphorylation (OXPHOS) to aerobic glycolysis. Endometriotic cells favor glycolytic metabolism over mitochondrial OXPHOS to produce essential energy for cell survival. Hypoxia, a common feature of the endometriosis environment, is a key player in this metabolic conversion, which may lead to glucose transporter overexpression, pyruvate dehydrogenase kinase 1 (PDK1) and lactate dehydrogenase kinase A (LDHA) activation, and pyruvate dehydrogenase complex inactivation. Evading mitochondrial OXPHOS mitigates excessive generation of reactive oxygen species (ROS) that may trigger cell death. Therefore, the coinactivation of LDHA and PDK1 can induce the accumulation of mitochondrial ROS by converting energy metabolism to mitochondrial OXPHOS, causing endometriotic cell death. Metabolic pattern reconstruction in endometriotic lesions is a critical factor in cell survival and disease progression. One therapeutic strategy that may avoid hormone manipulation is focused on mitigating metabolic changes that have been detected in cells/tissues from women with endometriosis.

**Lay summary:**

The most commonly used medical therapies for endometriosis have contraceptives and other side effects associated with hormone suppression and are therefore unsuitable for women desiring pregnancy. One therapeutic strategy that may avoid hormone manipulation is focused on changing metabolic profiles that have been detected in cells/tissues from women with endometriosis. Endometriotic cells favor glycolytic metabolism over mitochondrial oxidative phosphorylation (OXPHOS) to produce essential energy for cell growth. Furthermore, the metabolic conversion from mitochondrial OXPHOS to aerobic glycolysis suppresses cell death through the reduced generation of reactive oxygen species (ROS). This unique metabolic feature of endometriosis is important for cell survival and disease progression. Thus, changing the specific metabolic switch may increase mitochondrial ROS production, causing severe oxidative stress and cell death. This review describes new treatments by changing the metabolic profiles of endometriosis.

## Introduction

Endometriosis is an estrogen-dependent, chronic inflammatory condition that contains tissue that resembles an endometrium with one or more of the following: stromal fibroblasts, epithelial cells, immune cells, and nerves and vascular/perivascular cells in sites outside the uterine cavity (Zondervan *et al.* 2020, Saunders & Horne 2021). Moreover, it affects approximately 10% of all reproductive-aged women and is associated with pain and infertility (Hughes *et al.* 2015, Zondervan *et al.* 2020, Saunders & Horne 2021). The treatment choice will depend on age at diagnosis, disease stage, the patient’s symptoms, priorities and expectations, reproductive plans, safety, adverse effects incidence, tolerability, and cost (Ferrero *et al.* 2018). Medical endometriosis therapy should consider pain symptom control and postoperative recurrence prevention within the framework of long-term therapeutic strategies (Ferrero *et al.* 2018). However, the available drugs (e.g. combined oral contraceptive pills, progestins, danazol, and gonadotropin-releasing hormone (GnRH) analogs) suppress ovarian function and are not curative (Hughes *et al.* 2015, Ferrero *et al.* 2018). Thus, patients with endometriosis urgently need long-term nonhormonal therapy without affecting fertility.

Endometriosis exists in a unique inflammatory microenvironment characterized by hormonal imbalance, hypoxia, and oxidative stress (McKinnon *et al.* 2016, Ito *et al.* 2017, Lin *et al.* 2018). Endometriotic cells undergo genetic, epigenetic, and metabolic alterations to overcome many obstacles (e.g. adaptation and survival to harsh environments, evasion of immune defenses, and invasion of adjacent tissues; Koninckx *et al.* 2019). These microenvironmental changes can enhance the survival of endometriotic cells through several main pathways (e.g. phosphatidylinositol 3-kinase (PI3K)/protein kinase B (AKT)/mammalian target of rapamycin (mTOR), mitogen-activated protein kinases (MAPK; extracellular signal-regulated kinase (ERK)1/2, p38, and c-Jun NH2-terminal kinase (JNK)), and nuclear factor-kappaB (NF-κB) signaling pathways; McKinnon *et al.* 2016). These kinase pathways have been evaluated as effective targets for the treatment of other diseases, especially cancer (3), and can be potential candidates for personalized endometriosis therapy (McKinnon *et al.* 2016). However, the current generation of these targeted therapies can induce various adverse effects (McKinnon *et al.* 2016). Moreover, accumulating evidence shows that endometriotic cells may survive the hypoxic environment by upgrading their metabolic properties (Atkins *et al.* 2019). The metabolic shift between aerobic glycolysis and oxidative phosphorylation plays a major role in the development and progression of endometriosis, and the modification of their signaling pathways can be a viable target for therapeutic intervention (Liao *et al.* 2015, Kobayashi *et al.* 2021*a*). The review aims to discuss the survival mechanism of endometriosis in hypoxic and oxidative stress environments and provide future perspectives on nonhormone treatment based on metabolic shifts.

## Methods

### Search strategy and selection criteria

**Figure 1:**
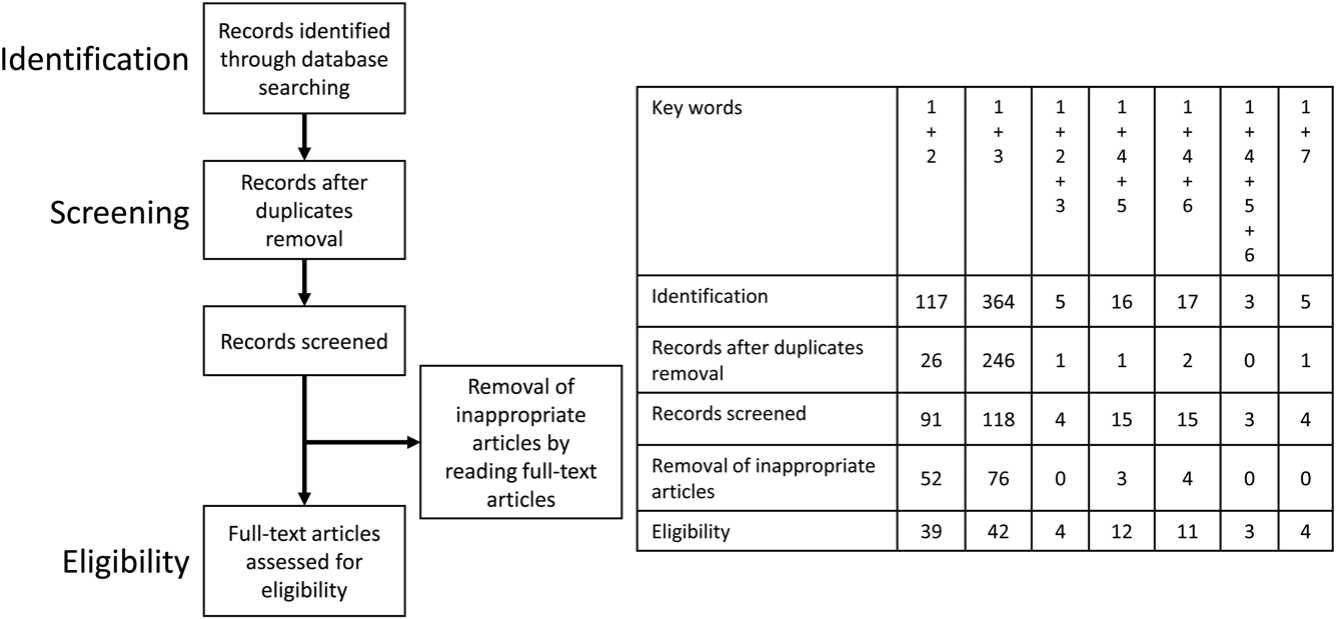
The number of articles identified by searching for keyword combinations. This figure shows the number of articles identified by keyword combinations and the number of records identified through database searching, records after duplicate removal, records screened, removal of inappropriate articles by reading full-text articles, and full-text articles assessed for eligibility. Keywords: 1, endometriosis; 2, hypoxia; 3, oxidative stress; 4, metabolism; 5, glycolysis; 6, oxidative phosphorylation; and 7, Warburg.

A computerized literature search was performed to identify relevant studies reported in the English language. The PubMed electronic databases published between January 2000 and March 2021 were searched, combining the keywords *endometriosis*, *hypoxia*, *oxidative stress*, *metabolism*, *glycolysis*, *oxidative phosphorylation*, and *Warburg*. The references of each article were searched to identify potentially relevant studies. Publications of original studies and review papers were included. Given the heterogeneity in the research theme, data from the studies were synthesized using a descriptive review design with narrative methods. Figure 1 shows that the first identification phase includes the records identified through database search. Terms in the titles and abstracts were focused in the first screening stage. During the second screening phase, duplicates were removed, and titles, abstracts, and full-text articles were read to remove inappropriate papers. The final eligibility phase included the full-text articles for analysis after excluding those for which detailed data cannot be extracted.

## Results and discussion

A review of the literature provides evidence that endometriotic cells may undergo metabolic change/adaptation to survive in extrauterine sites under conditions that may involve hypoxia and/or oxidative stress. The evidence is considered a shift in metabolic behavior under oxidative stress and hypoxia to inform the discussion of potential novel therapies. Here, three topics of endometriosis (i.e. oxidative stress and redox imbalance, hypoxic microenvironment, and metabolic reprogramming) will be discussed.

### Oxidative stress and redox imbalance

Several theories have been proposed to explain the etiology of endometriosis, which includes the theories on retrograde menstruation, coelomic metaplasia, endometrial stem/progenitor cells, bone marrow stem cells, lymphatic and vascular spread, embryonic remnant differentiation or induction, and iatrogenic implantation (Zubrzycka *et al.* 2015). The most widely accepted is the retrograde menstruation theory. Blood containing endometrial cells is refluxed through the fallopian tubes during menstruation (Vinatier *et al.* 2000). Hemoglobin releases heme iron and free iron when red blood cells are hemolyzed in the peritoneal cavity or endometriotic cysts (Kobayashi *et al.* 2009). Hemoglobin generates superoxide radicals (O_2_^−^) when converted to methemoglobin via the autoxidation reaction (Iwabuchi *et al.* 2015). Free iron also generates hydroxyl radicals (OH^−^), a powerful reactive oxygen species (ROS), through the Fenton reaction (Iwabuchi *et al.* 2015). Thus, endometriotic cells are always exposed to exogenous ROS, including superoxide anion, hydroxyl radical, and peroxynitrite (ONOO^−^). High ROS levels induce oxidative DNA damage, methylation, and epigenetic errors (Menezo *et al.* 2016). Oxidative stress caused by ROS is a potential factor involved in the pathogenesis of endometriosis and may play a role in the onset and progression of this disease (Menezo *et al.* 2016, Ito *et al.* 2017). However, excessive ROS generation is also a key factor leading to cell death. Several studies have evaluated the oxidant–antioxidant balance in the blood, peritoneal fluid, follicular fluid, and tissue environment of patients with endometriosis (Santanam *et al.* 2002, Muscoli *et al.* 2003, Oner-Iyidoğan *et al.* 2004, Matos *et al.* 2009, Liu *et al.* 2013, Bamm *et al.* 2017, Chen *et al.* 2019). The ROS levels in both serum and follicular fluid of the endometriosis group were significantly higher than those in both serum and follicular fluid of the control group (Liu *et al.* 2013). The conjugated diene/triene, malondialdehyde, and oxidized low-density lipoproteins are lipid oxidation biomarkers (Santanam *et al.* 2002, Bamm *et al.* 2017). The levels of these lipid peroxidation end products were increased in both peritoneal fluid and serum of patients with endometriosis (Santanam *et al.* 2002, Bamm *et al.* 2017). Furthermore, the antioxidant capacities (e.g. superoxide dismutase (SOD) activity) were increased in endometriosis (Oner-Iyidoğan 2004, Matos *et al.* 2009, Chen *et al.* 2019). ROS suppresses SOD production, but SOD expression is upregulated in endometriosis despite ROS overproduction (Muscoli *et al.* 2003). Antioxidants maintain cellular redox homeostasis by eliminating ROS and protecting cells from ROS-induced damage (Chen *et al.* 2019). Thus, endometriotic cells can survive with oxidative stress exposure.

### Hypoxic microenvironment

Endometrial fibroblasts are decidualized during pregnancy, allowing placenta formation and embryo implantation (Rytkönen *et al.* 2020). Placental tissue may have evolved mechanisms to tolerate hypoxic environments by expressing hypoxia-related genes such as hypoxia-inducible factor-1alpha (HIF-1α), vascular endothelial growth factor (VEGF), and transforming growth factor-beta1 (TGF-β1; Duzyj *et al.* 2018). Ectopic endometriotic cells also appear to inherit this property. Ectopic endometrial cells face severe hypoxic stress, but hypoxia plays a vital role in promoting pathological processes to facilitate endometriosis development (Lin *et al.* 2018, Lee *et al.* 2019, Wu *et al.* 2019). Under a hypoxic condition, cells undergo genetic and epigenetic modifications and evolve several survival processes, including steroidogenesis, inflammation, immune dysfunction, angiogenesis, epithelial–mesenchymal transition (EMT), and mesothelial–mesenchymal transition (MMT; Wu *et al.* 2019). The complex gene regulatory networks driven by the interplay between a hypoxic microenvironment and endometriotic cells allow endometriotic cells to survive (Wu *et al.* 2019). The effects induced by hypoxia are orchestrated by HIFs that regulate the expression of numerous genes, including VEGF, TGF-β1, PI3K/AKT, Wnt/β-catenin, and Notch (Laschke & Menger 2012, Wilson 2018, Rytkönen *et al.* 2020). Genes related to classical hypoxia pathways (e.g. HIF-1α, VEGF, and TGF-β1) have been extensively studied in endometriotic cells (Laschke & Menger 2012, Rytkönen *et al.* 2020) and adjacent peritoneal mesothelial cells (Wilson 2018). A hypoxic microenvironment stimulates endometriotic stromal cells (Dai *et al.* 2019) and peritoneal mesothelial cells (Lin *et al.* 2018) to produce and stabilize HIF-1α and promote the activation of TGF-β1/Smad and VEGF signal transduction pathways, contributing to increased cellular invasiveness, adhesiveness, cell survival, EMT, MMT, adhesion and fibrosis formation, and reduced apoptotic potential (Kasvandik *et al.* 2016). Endometriosis can be caused by local changes in tissues under the influence of oxidative stress and associated hypoxia. In addition, hypoxia has recently been emphasized to upregulate genes associated with glycolysis as described in the next section.

### Metabolic reprogramming

**Figure 2:**
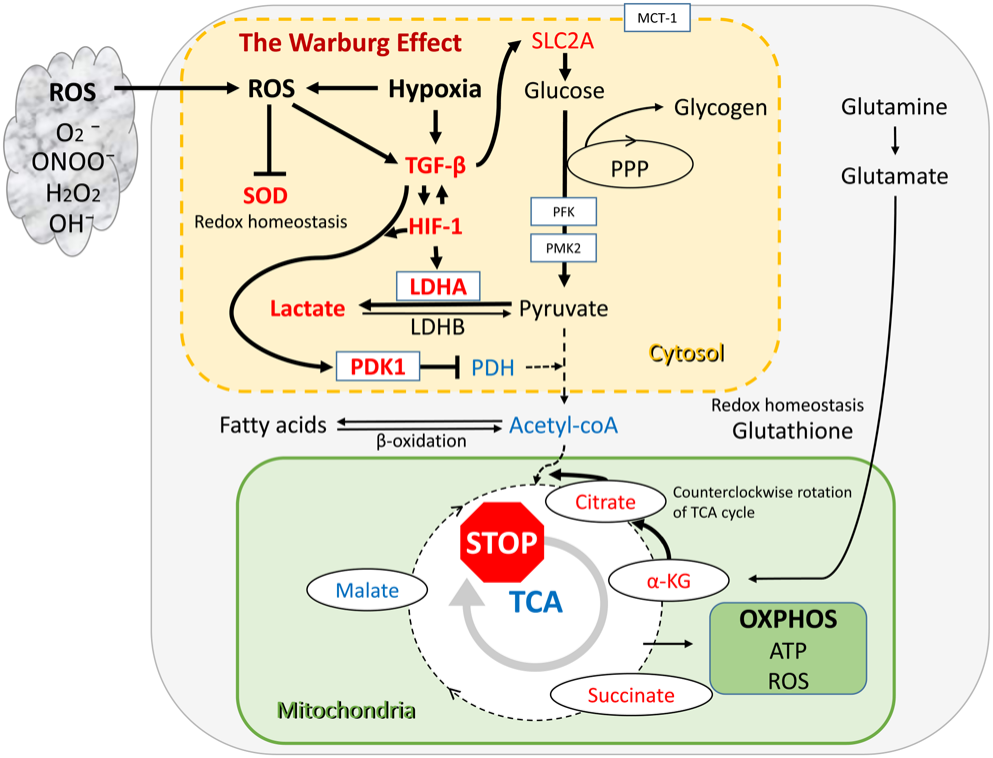
Glycolysis and mitochondrial metabolism in endometriosis. *Colored boxes* indicate major metabolic pathways: aerobic glycolysis (*yellow box*) and the TCA cycle/OXPHOS (*green box*). *Red letters* indicate increased genes, gene transcripts, enzymes, and metabolites; *blue letters* indicate reduced expression.

The metabolic properties of endometriosis for energy acquisition are discussed in this section. In general, glycolytic conversion of glucose or fructose into adenosine 5′-triphosphate (ATP) generates energy to enable cell survival and growth (Fig. 2). Cells utilize aerobic glycolysis to derive energy from the conversion of glucose to pyruvate and then lactate, regardless of oxygen availability (Vander Heiden *et al.* 2009). Aerobic glycolysis produces only two ATP per one glucose molecule, whereas additional 36 ATP molecules from one glucose molecule are produced through the tricarboxylic acid (TCA) cycle and the OXPHOS machinery (Vander Heiden *et al.* 2009). Aerobic glycolysis is an inefficient way to generate ATP, but it is a simple mechanism with a high ATP production rate. Aerobic glycolysis is activated by the stimulation of glycolytic enzymes such as glucose transporter (GLUT; McKinnon *et al.* 2014, Di Tucci *et al.* 2018), phosphofructo-2-kinase/fructose-2,6-biphosphatase 3 (PFKFB3; Yi *et al.* 2019), pyruvate kinase M2 (PKM2; Tamada *et al.* 2012), pyruvate dehydrogenase kinase 1 (PDK1; Dunford *et al.* 2011), pyruvate dehydrogenase (PDH; Dunford *et al.* 2011), lactate dehydrogenase A (LDHA; Miao *et al.* 2013), and monocarboxylate transporter 1 (MCT-1; Halestrap 2012; Fig. 2; glycolytic pathways are surrounded by a yellow square). PFKFB3, as a key enzyme of glycolysis, positively regulates the glycolysis process (Yi *et al.* 2019). PKM2 is a final rate-limiting glycolysis enzyme and supports anabolic metabolism (Tamada *et al.* 2012). Pyruvate is converted to acetyl-coenzyme A (CoA), which is catalyzed by the PDH complex (Lapel * et al.* 2017). PDK1 is an enzyme that phosphorylates and deactivates PDH (Dunford *et al.* 2011). In addition, LDHA catalyzes the conversion of pyruvate to lactate and is considered a key checkpoint of anaerobic glycolysis (Miao *et al.* 2013). MCT-1 facilitates the rapid intracellular and extracellular transport of monocarboxylates (e.g. pyruvate, lactate, and the ketone bodies; Halestrap 2012). Fatty acids are transported to the mitochondria and then metabolized to acetyl-CoA by β-oxidation, which feeds the TCA cycle. Acetyl-CoA is the key starting point of the mitochondrial TCA cycle and an essential fuel for ensuring OXPHOS (Fig. 2; mitochondrial oxidative phosphorylation pathways are surrounded by a green square). However, stimulation of pyruvate flux into the mitochondrial oxidative metabolism increases ROS production, an inherent byproduct of oxidative metabolism, leading to impaired cell survival. Thus, a shift in metabolism from glycolysis to the TCA cycle/OXPHOS has not only the advantage of high energy production but also the drawback of ROS overproduction.

Endometriotic cells have been shown to reprogram metabolism pathways in response to various hypoxic and oxidative stress to fuel cell survival (Dunford *et al.* 2011, Young *et al.* 2014, 2016, Kasvandik *et al.* 2016, Lee *et al.* 2019). Endometriotic cells can induce metabolic conversion from oxidative phosphorylation to aerobic glycolysis to suppress ROS-mediated apoptosis. Four major steps are involved in cell metabolism: glucose uptake, glycolytic enzyme activation, lactate production and accumulation, and changes in mitochondrial function. For each step, the latest information on the metabolic alterations in endometriosis is summarized.

#### Enhanced glucose uptake

Glycolysis begins with glucose uptake through solute carriers of the GLUT family (McKinnon *et al.* 2014). Solute carrier family 2 (SLC2A) gene encodes an integral plasma membrane glycoprotein, GLUT. The expression of SLC2A3 (GLUT3), SLC2A4 (GLUT4), and SLC2A5 (GLUT5) genes and proteins in endometriotic tissues was significantly higher than that in eutopic tissues (McKinnon *et al.* 2014). HIF1A gene expression was higher in endometriotic lesions than in eutopic endometrium, and the HIF1A and SLC2A1 gene expression levels in the adjacent peritoneum of endometriotic lesions were higher than those in women without the disease (Di Tucci *et al.* 2018). An* in vitro* study showed that exposure of peritoneal mesothelial cells to TGF-β1 increased HIF1A and SLC2A1 mRNA expression (Di Tucci *et al.* 2018). Cellular glucose uptake by GLUTs is activated via the upregulation of TGF-β expression (McKinnon *et al.* 2014). Enhanced glucose uptake as a result of increased HIF-1 and TGF-β1 expression is a hallmark of endometriosis. Therefore, endometriosis causes metabolic reprogramming by increasing glucose uptake via the GLUT family (Fig. 2, yellow box).

#### Increased glycolytic capacity and lactate production

Ectopic endometriotic cells exhibit more hypoxia than their eutopic counterparts (Lee *et al.* 2019). Some researchers compared tissue, peritoneal fluid, follicular fluid, and blood samples from patients with endometriosis to controls and showed significant changes in glycolytic pathway-specific genes and their transcripts (HIF-1, TGF-β, LDHA, PDK1, PDH, and SOD) and metabolites (glucose and lactate), indicating a distinct glucose metabolic signature (Qi *et al.* 2014, Young *et al.* 2014, 2016, Marianna *et al.* 2017, Horne *et al.* 2019). Lactate, an essential glycolysis product, is a major metabolic fuel, energy source, and gluconeogenic precursor. Lactate concentration was positively correlated with TGF-β1 in peritoneal fluid, and both of which were significantly higher in women with endometriosis than in women without endometriosis (Qi *et al.* 2014, Young *et al.* 2014, 2016, Horne *et al.* 2019). TGF-β1 can induce the metabolic conversion of glucose to lactate in the endometriotic lesions and adjacent peritoneum, possibly through hypoxia-induced HIF-1α expression (Young *et al.* 2014, 2016). Moreover, hypoxia-induced PDK1 upregulation and increased lactate production and oxygen consumption rate in ectopic endometrial stromal cells compared to normal endometrial stromal cells (Lee *et al.* 2019). This is thought to be because PDK1 suppressed the conversion of pyruvate to acetyl-CoA through the inhibition of PDH activity (Dunford *et al.* 2011). Exposure of mesothelial cells to TGF-β1 increased the production of mRNAs encoded by glycolysis-associated genes, namely, PDK1 and LDHA (Young *et al.* 2014). Glycolysis-related gene LDHA was more highly expressed in endometriotic lesions than in a eutopic endometrium (Young *et al.* 2014). Furthermore, follicular fluid in patients with endometriosis had lower glucose levels and higher levels of lactate, pyruvate, and VEGF than those in follicular fluid in control participants (Marianna *et al.* 2017, Pocate-Cheriet *et al.* 2020). Increased glucose uptake and consumption and accumulation of lactate were common features of endometriotic cells (Qi *et al.* 2014). Lactate has been reported to be proangiogenic (Hunt *et al.* 2008). Although no experimental data using endometriotic cells exist, lactate stimulates VEGF production by tumor and endothelial cells, leading to enhanced migration and resulting in lactate-induced angiogenesis (Hirschhaeuser *et al.* 2011, Marianna *et al.* 2017). Altogether, endometriotic cells have an increased glycolytic flux, which depends on the overexpression of glycolysis-related genes or their transcripts (HIF-1α, TGF-β, GLUT, LDHA, and PDK1), resulting in lactate overproduction and accumulation (Qi *et al.* 2014, Young *et al.* 2014, 2016, Marianna *et al.* 2017, Horne *et al.* 2019). The metabolic switch of increased glycolysis in endometriosis is thought to be driven primarily by TGF-β and HIF-1α (Fig. 2, yellow box).

#### Metabolic conversion from TCA cycle/OXPHOS to aerobic glycolysis

Activation of aerobic glycolysis raises two possibilities. First, pyruvate is channeled into the mitochondria and converted to acetyl-CoA, and then enters the TCA cycle. Hypoxia-induced PDK1 expression results in decreased PDH activity, suppresses the conversion of pyruvate to acetyl-CoA, and accumulates pyruvate (Dunford *et al.* 2011, Young *et al.* 2014, 2016, Kasvandik *et al.* 2016, Lee *et al.* 2019). Second, LDHA promotes the conversion of pyruvate to lactate and suppresses the production of acetyl-CoA. Therefore, the conversion of pyruvate to acetyl-CoA in endometriosis may be suppressed by increased LDHA and PDK1 activity and decreased PDH activity (Young *et al.* 2014, 2016, Kasvandik *et al.* 2016) (Fig. 2, green box).

Next, reports on the concentrations of intermediate metabolites involved in glycolysis and the TCA cycle from body fluid samples in patients with endometriosis and controls were summarized. Endometriosis patients showed greater changes in levels of metabolites (e.g. glucose, lactate, citrate, alpha-ketoglutarate, succinate, and malate) compared to controls. Serum (Dutta *et al.* 2012) and follicular fluid (Marianna *et al.* 2017, Karaer *et al.* 2019) samples from patients with endometriosis showed elevated lactate and succinate levels and reduced glucose levels compared to controls. Metabolomics analysis revealed that citrate, alpha-ketoglutarate (α-KG), and succinate were elevated in endometriosis (Jana *et al.* 2013) whereas malate was decreased (Atkins *et al.* 2019). The cause for the elevated citrate, α-KG, and succinate in endometriosis was considered. In general, glycolysis, glutaminolysis, or fatty acid β-oxidation provides the energy and macromolecules required for cell survival. For example, cancer patients show distinctly altered metabolism involved in glycolysis, TCA cycle, glutaminolysis, and fatty acid metabolism (Zhu *et al.* 2017). In the event of an energy crisis, the glutaminolysis involved in the conversion of glutamine to α-KG is activated to sustain energy metabolism (DeBerardinis *et al.* 2007). Glutaminolysis stimulates a pathway in which citrate was formed from α-KG through reductive carboxylation of isocitrate dehydrogenase (Wise *et al.* 2011). Therefore, endometriotic mitochondria can produce large amounts of α-KG and citrate (Fig. 2, green box). Furthermore, glutaminolysis supports the production of glutathione, a major player in maintaining redox homeostasis (Wise & Thompson 2010). Thus, endometriosis can adapt to a unique environment by suppressing oxidative stress and enhancing its antioxidant capacity.

Surprisingly, despite survival in harsh environments, mitochondrial energy production and metabolism are reduced in endometriotic tissue compared to normal endometrial tissue (Atkins *et al.* 2019). Peritoneal mesothelial cells adjacent to endometriotic lesions also exhibited significantly higher glycolysis, increased lactate production, and lower mitochondrial respiration compared to those from women without the disease (Horne *et al.* 2019). These data suggest that endometriotic cells and adjacent peritoneal mesothelial cells are characterized by TCA cycle/OXPHOS arrest and metabolic shift to aerobic glycolysis. Endometriosis can alter cellular metabolism and can strategically reduce energy production to avoid excessive mitochondrial ROS production. The electron-producing oxidative pathway appears to be stopped in endometriosis, resulting in the lack of energy production.

Does this suggest mitochondrial dysfunction? The metabolic shift from OXPHOS to glycolysis is known as the Warburg effect and is a characteristic of many cancers (Kasvandik *et al.* 2016, Liberti & Locasale 2016, Atas *et al.* 2020). This mechanism can be driven by the TGF-β1–HIF-1α–PDK–PDH–LDHA system (Kim *et al.* 2006, Young *et al.* 2016, Wang *et al.* 2019, Atas *et al.* 2020). HIF-1 and TGF-β increased LDHA expression, promoted lactate production from pyruvate, and inhibited acetyl-CoA production from pyruvate through PDH deactivated by PDK1, consequently reducing mitochondrial energy production and ROS generation (Liao *et al.* 2015, Wang *et al.* 2019). The advantage of the Warburg effect is to suppress ROS overproduction, activate the survival signal of endometriotic cells, and thus prevent cell death (Kobayashi *et al.* 2021*b*). Like cancer cells, endometrial cells may shift energy metabolism from OXPHOS to aerobic glycolysis, suppress ROS production, and then promote survival (Liao *et al.* 2015, Kobayashi *et al.* 2021*b*). Alterations in the metabolic phenotype of endometriotic cells and adjacent peritoneal mesothelial cells are considered adaptations of endometriosis to the microenvironment rather than mitochondrial dysfunction.

### Nonhormonal endometriosis treatment

This section discusses therapies that may alter energy metabolism, including the so-called *Warburg effect*. Treatment strategies for endometriosis have been divided into three categories: (1) glucose uptake suppression, (2) aerobic glycolysis suppression, and (3) metabolic switch from aerobic glycolysis to OXPHOS. Not all of the drugs described below have yielded promising preclinical outcomes for endometriosis. Some drugs that have been tested as therapies in preclinical models involving cancer cells that also exhibit altered metabolism have been considered (Table 1).

**Table 1:** Summary of drugs or therapeutics tested in endometriosis and other models. This table includes target protein/metabolite, mechanism of action,* in vitro*/*in vivo*/animal experiments, results, and references.

Target protein/metabolite	The mechanism of action	*In vitro*/*in vivo*/animal experiments	Results	References
(1) Suppression of glucose uptake				
Genistein	A natural isoflavone	*In vitro*/xenograft mouse models; hepatocellular carcinoma cells	Genistein suppressed aerobic glycolysis and induced hepatocellular carcinoma cell death	Li *et al*. (2017*b*)
Genistein, phlorizin, ritonavir, indinavir, STF-31, and WZB117	A natural isoflavone; glucose transporter (GLUT and SGLT) inhibitors; HIV protease inhibitor	*In vivo*/*in vivo*/mouse model; PBMCs of patients with ulcerative colitis	The HIV protease inhibitor ritonavir suppressed glucose uptake to improve ulcerative colitis	Jodeleit *et al*. (2018)
SLC2A*	Glucose transporter	Human tissue samples	Glucose transporter SLC2A expression in ectopic endometriotic lesions is significantly higher than in eutopic endometrial tissue	McKinnon *et al*. (2014)
GZFLC*	A classic Chinese medicinal formula	Rat endometriosis model	GZFLC suppressed the expression levels of TGF-β1, GLUT4, and VEGF and inhibited the development of endometriosis	Zhou *et al*. (2018)
Atorvastatin and resveratrol*	Statin: inhibitors of hydroxymethylglutaryl-CoA reductase	Female Wistar rats/the experimental endometriosis	Effects of atorvastatin and resveratrol against the experimental endometriosis; evidence for glucose and monocarboxylate transporters	Bahrami *et al*. (2021)
(2) Suppression of aerobic glycolysis				
Genetic ablation HK2	A family of ubiquitous exose-phosphorylating enzymes that prime glucose for intracellular utilization	Mouse models; hepatocellular carcinoma/colorectal cancer/glioblastoma, etc.	Genetic ablation of HK2 inhibited tumor growth	Ciscato *et al.* (2021)
(E)-1-(pyridin-4-yl)-3-(quinolin-2-yl)prop-2-en-1-one (PFK15)	Enzymes related to glycolysis; inhibitors of PFKFB3; glycolysis blockage by targeting PFKFB3	*In vitro*/*in vivo*/mouse models; head and neck squamous cell carcinoma	Targeting aerobic glycolysis with PFKFB3 inhibitors suppressed tumor growth and metastasis, providing a promising strategy for cancer treatment	Li *et al.* (2017*a*)
Benserazide: inhibitors of PKM2	PKM2 is an enzyme that generates pyruvate and ATP in the glycolytic pathway	*In vitro*/*in vivo*; melanoma	Benserazide blocked PKM2 enzyme activity, leading to inhibition of aerobic glycolysis; benserazide inhibited tumor cell proliferation, colony formation, invasion, and migration* in vitro* and* in vivo* models	Zhou *et al.* (2020)
Inhibitor of HSF1: KRIBB11*	A transcription factor that is rapidly induced after temperature stress and binds heat shock promoter elements	*In vitro*/*in vivo*/mouse models; endometriotic epithelial cell line (11Z) and human ESC	HSF1 promoted endometriosis development and glycolysis by upregulating PFKFB3 expression; the HSF1 inhibitor KRIBB11 abrogated endometriosis progression* in vitro* and* in vivo*	Wang *et al.* (2021)
(3) Metabolic switch from aerobic glycolysis to OXPHOS				
DCA	DCA is an anticancer agent that can reverse the glycolytic phenotype in cancer cells; a pyruvate analog; a prototypical PDK inhibitor	Several cancers	DCA inhibits mitochondrial PDK, shifted metabolism from glycolysis to glucose oxidation, decreased mitochondrial membrane potential, and increased mitochondrial H_2_O_2_; DCA decreased proliferation, induced apoptosis, and inhibited tumor growth; the orally available DCA is a promising selective anticancer agent	Bonnet *et al.* (2007)
DCA		*In vitro*/*in vivo*/rat models; breast cancer	DCA has antiproliferative properties in addition to promoting apoptosis	Sun *et al.* (2010)
DCA		*In vitro*/*in vivo*/mouse models; multiple myeloma	DCA may be effective in multiple myeloma patients with an activated aerobic glycolytic pathway	Sanchez *et al.* (2013)
DCA		Several cancer models; clinical administration in cancer therapy	Coadministration of DCA with conventional chemotherapy, radiotherapy, other drugs, or natural compounds may be promising for effective cancer therapy	Tataranni & Piccoli (2019)
Three glycolysis inhibitors: DCA, 2-deoxyglucose, or 3-promopyruvate		*In vitro*; hepatocellular carcinoma HepG2 cells	The chemotherapeutic agent and glycolysis inhibitors induced oxidative stress-associated damage in HepG2 cells.	Korga *et al.* (2019)
DCA		A phase 1 study in patients with advanced solid tumors	The phase 1 study was undertaken to assess the safety, recommended dose, and pharmacokinetic profile of oral DCA in patients with advanced solid tumors.	Chu *et al.* (2015)
DCA		An openlabel phase II trial	The clinical trial determined the response rate, safety, and tolerability of oral DCA in patients with metastatic breast cancer and advanced-stage nonsmall cell lung cancer. Patients with previously treated advanced cancer did not benefit from oral DCA	Garon *et al.* (2014)
DCA		A pilot phase 2 study in patients with multiple myeloma	The pharmacokinetic profile for DCA varied from patient to patient, and the overall response rate for multiple myeloma was low	Tian *et al.* (2019)
DCA*		*In vitro;* ectopic endometriotic stromal cells	The PDK1 expression was upregulated in ectopic stromal cells through hypoxia-induced signals; inhibition of PDK1 activity by treatment with DCA-induced ectopic stromal cell death	Lee *et al.* (2019)
DCA*		*In vitro*/*in vivo*/mouse models; endometriosis	Human peritoneal mesothelial cells (HPMC) in women with endometriosis exhibited metabolic conversion from OXPHOS to aerobic glycolysis due to reduced enzymatic activity of PDH compared to HPMC in disease-free women; TGF-β1 is believed to be responsible for this abnormal phenotype; treatment of endometriosis HPMC with DCA normalizes metabolism and suppresses the proliferation of ESC; oral DCA reduced endometriosis lesion size in a mouse model	Horne *et al.* (2019)
DCA		Sepsis model: *Drosophila melanogaster* model of surviving sepsis infected with *Staphylococcus aureus*	DCA treatment was associated with improved lifespan of sepsis survivors	Bakalov *et al.* (2020)
IQ	A sesquiterpene quinone isolated from the marine sponge *Smenospongia cerebriformis*; PDK1 inhibitor	Human and murine cancer cells, such as A549, DLD-1, RKO, and LLC cells	A novel candidate for anticancer therapeutics that act via the inhibition of PDK1 activity	Kwak *et al.* (2020)
*Caesalpinia sappan* L. (family Leguminosae)*	A herbal medicinal product used to treat gynecological symptoms, including amenorrhea; PDK1 inhibitor	*In vitro*; endometriotic cells	*C. sappan* inhibited lactate production and phosphorylation of PDH by reducing the expression of PDK1; a novel drug candidate for treating endometriosis by inhibiting aerobic glycolysis and inducing ROS-mitochondria-mediated apoptotic cell death	Kim *et al.* (2021)
FX11	Specific LDHA inhibitor: a small-molecule inhibitor	*In vitro*/human lymphoma and pancreatic cancer xenografts	FX11-induced significant oxidative stress and cancer cell death	Le *et al.* (2010)
N-hydroxy-2-carboxy-substituted indole compounds	Specific LDHA inhibitor: a small-molecule inhibitor	*In vitro* NMR experiments	Functional analysis of synthesized LDHA inhibitors	Granchi *et al.* (2011)
Inhibition of LDHA by either RNA interference or pharmacological agents	Inhibition of LDHA by either RNA interference or pharmacological agents	*In vitro*/*in vivo*; several cancer cells.	Review of inhibition of LDHA by either RNA interference or pharmacological agents block tumor progress* in vivo*	Oermann *et al.* (2012)
Inhibition of LDHA by either RNA interference or pharmacological agents	Inhibition of LDHA by either RNA interference or pharmacological agents	*In vitro*/*in vivo*; cancers including breast cancer and hepatocellular carcinoma	Review of inhibition of LDHA can block tumor growth, maintenance, and progression* in vitro* and* in vivo*	Miao *et al.* (2013)
shRNA-mediated knockdown of LDHA	Inhibition of LDHA by either RNA interference or pharmacological agents	*In vitro*; breast cancer MDA-MB-435 cells	shRNA-mediated knockdown of LDHA resulted in elevated mitochondrial ROS production and a concomitant decrease in cell proliferation and motility in breast cancer MDA-MB-435 cells	Arseneault *et al.* (2013)
Inhibition of LDHA by either RNA interference*	Inhibition of LDHA by either RNA interference	Immunohistochemistry of human endometriosis samples; *in vitro*.	Hypoxia treatment induced the expression of LDHA; silencing of LDHA expression displayed an impairment of mitochondrial function and promoted apoptosis while inhibiting migration and glycolysis	Zheng *et al.* (2021)
None	Schizophrenia	Experiments with schizophrenia brain	A significant increase in lactate in schizophrenia brain	Pruett and Meador-Woodruff (2020)
None	Autoimmune disease	Animal studies	Pro-inflammatory signals in autoimmune disease induced metabolic reprogramming, characterized by a shift to aerobic glycolysis	Kornberg (2020)

*Results of preclinical studies on endometriosis.

DCA, dichloroacetate; ESC, endometrial stromal cells; FX11, 3-dihydroxy-6-methyl-7-(phenylmethyl)-4-propylnaphthalene-1-carboxylic acid; GZFLC, Gui-Zhi-Fu-Ling capsules; HK2, hexokinase 2; HSF1, heat shock factor 1; IQ, ilimaquinone; PBMCs, peripheral blood mononuclear cells; PFKFB3, phosphofructokinase-2/fructose-2,6-bisphosphatase 3; PKM2, pyruvate kinase isozyme; SLC2A, solute carrier family 2.

#### Glucose uptake suppression

The potent GLUT inhibitors can attenuate glycolysis and suppress the growth of various cancer cells (Reckzeh *et al.* 2019). GLUT and SGLT inhibitors include genistein, phlorizin, ritonavir, indinavir, STF-31, and WZB117 (Jodeleit *et al.* 2018). Genistein downregulates HIF-1α, inactivating GLUT1 and HK2 to suppress aerobic glycolysis (Li *et al.* 2017*b*). GLUTs were identified as off-target molecules of the HIV protease inhibitor ritonavir (Jodeleit *et al.* 2018). They exert antitumor effects by targeting GLUT1 via inhibiting glucose uptake in tumor cells. Recently, the glucose uptake inhibitors, which target GLUT isoforms, have also been studied for endometriotic cells. In particular, GLUT inhibitors may be an attractive target for the nonhormone-based treatment of endometriosis (McKinnon *et al.* 2014). The mRNA levels of GLUT1/3 and MCT1/4 were decreased in atorvastatin and resveratrol sole and simultaneous-treated groups in experimental endometriosis models (Bahrami *et al.* 2021). Atorvastatin did not cause significant changes during the glucose tolerance test, but coadministration of atorvastatin and resveratrol suppressed glycolysis and neovascularization (Bahrami *et al.* 2021). The simultaneous administration of atorvastatin and resveratrol can inhibit endometriosis development (Bahrami *et al.* 2021). Gui-Zhi-Fu-Ling capsules, a classic Chinese medicinal formula, may have benefits in inhibiting endometriosis development through the suppression of the expression levels of TGF-β1, GLUT4, and VEGF in a rat endometriosis model (Zhou *et al.* 2018). Thus, inhibition of glucose uptake may be promising therapeutic targets for endometriosis (McKinnon *et al.* 2014).

#### Aerobic glycolysis suppression

Glycolytic enzyme inhibitors such as hexokinase (HK), phosphofructokinase (PHK), and PKM2 have been preclinically studied in cancer treatment.

*HK:* Hexokinase, an exose-phosphorylating enzyme for aerobic glycolysis, is overexpressed in many tumor cells (Ciscato *et al.* 2021). Treatments with 2-deoxy-d-glucose, 3-bromopyruvate, or lonidamine inhibit the key enzyme hexokinase of glycolysis, and genetic ablation of hexokinase 2 inhibits tumor growth in mouse models (Ciscato *et al.* 2021).
*PFK:* Phosphofructokinase-1 (PFK1), a primary glycolysis enzyme, is involved in the conversion of fructose-6-phosphate to fructose-1,6-bisphosphate (Li *et al.* 2017*a*). (E)-1-(pyridin-4-yl)-3-(quinolin-2-yl)prop-2-en-1-one (PFK15) was developed as a selective antagonist of PFK–PFKFB3 (Li *et al.* 2017*a*). PFK15 has been demonstrated to be effective in treating head and neck squamous cell carcinoma in xenograft mouse models (Li *et al.* 2017*a*). Furthermore, the PFKFB3 expression in endometriotic cells is known to be upregulated by heat shock factor 1 (HSF1; Wang *et al.* 2021). In addition, Wang *et al.* (2021) showed that the HSF1 inhibitor KRIBB11 suppressed endometriosis progression in a mouse model.
*PKM2:* The M2 splice isoform of PKM2 eventually produces pyruvate and releases energy. High PKM2 activity is associated with glycolytic capacity and tumor growth and metastasis (Zhou *et al.* 2020). Suppression of PKM2 expression attenuated cancer cell growth via modulating immunometabolism (Zhou *et al.* 2020).

Suppression of the aerobic glycolytic pathway may become a new target for endometriosis treatment, but studies are still in their infancy (Wang *et al.* 2021).

#### Metabolic switch from aerobic glycolysis to OXPHOS

The reversal of metabolism from OXPHOS to glycolysis, a metabolic characteristic of cancer cells, may be a therapeutic strategy that induces cell death through ROS overproduction by activating mitochondrial energy metabolism. The conversion of pyruvate to acetyl-CoA needs to be accelerated to reach that goal. PDH is essential for shuttling pyruvate into the mitochondria and fueling the TCA cycle. PDH activity is inhibited by PDK, and PDK inhibitors may help in activating PDH enzymatic activity. In addition, dichloroacetate (DCA) is a small-molecule pyruvate-mimetic PDK inhibitor (Bonnet *et al.* 2007). DCA promotes oxidative metabolism from anaerobic glycolysis to mitochondrial OXPHOS through PDH activation by PDK1 inhibition (Sun *et al.* 2010, Horne *et al.* 2019, Tataranni & Piccoli 2019). This drug was shown to reverse the PDK-induced glycolytic phenotype (Bonnet *et al.* 2007, Tataranni & Piccoli 2019). DCA suppressed the growth of some tumors in the field of cancer, and several preclinical studies have been reported (Bonnet *et al.* 2007, Sun *et al.* 2010, Sanchez *et al.* 2013, Korga *et al.* 2019, Tataranni & Piccoli 2019). DCA can induce cell death via excess ROS produced by OXPHOS (Tataranni & Piccoli 2019). Therefore, this drug is a promising adjuvant chemotherapeutic agent as an oxidative stress enhancer (Korga *et al.* 2019). For example, DCA is potentially effective against multiple myeloma in animal models (Sanchez *et al.* 2013). In line with this theory, novel clinical DCA studies in cancer therapy are underway (Garon *et al.* 2014, Chu *et al.* 2015, Tian *et al.* 2019). The phase 1 study evaluated the safety, tolerability, recommended dose, pharmacokinetics, and pharmacodynamics of oral DCA in patients with advanced solid tumors (Chu *et al.* 2015). DCA produced side effects, including neurotoxicity. The open-label phase II trial determined the response rate, safety, and tolerability of oral DCA in patients with metastatic breast cancer and advanced-stage nonsmall cell lung cancer (Garon *et al.* 2014). However, oral DCA did not confer a clinical benefit in patients with previously treated advanced cancer. In addition, the pharmacokinetic profile for DCA varied from patient to patient, and the overall response rate was low in patients with multiple myeloma (Tian *et al.* 2019). PDK is a druggable target and may pave the way for further approaches to cancer.

Preclinical studies have shown that selective PDK inhibition suppresses the progression of endometriosis in animal models (Horne *et al.* 2019, Lee *et al.* 2019). *In vitro* and* in vivo* studies showed that DCA reduced lactate secretion and suppressed endometrial stromal cell proliferation in coculture experiments with endometrial stromal cells and peritoneal mesothelial cells (Horne *et al.* 2019). In addition, DCA decreased the oxygen consumption rate of ectopic endometrial stromal cells (Lee *et al.* 2019). Oral DCA administration decreased peritoneal fluid lactate concentration and lesion size in a mouse model of experimental endometriosis (Horne *et al.* 2019). Aerobic glycolysis mediates growth promotion and resistance to apoptosis of endometriotic cells, and a metabolic shift from glycolysis to OXPHOS is considered a promising therapeutic endometriosis strategy (Kim *et al.* 2021). A single-arm study has begun to determine whether DCA therapy is an effective and acceptable treatment for endometriosis-related pain (Leow *et al.* 2021). This study provides a rationale for targeting metabolic shifts as a nonhormonal therapy for women with endometriosis.

In addition, some reports on PDK1 inhibitors such as ilimaquinone (IQ; Kwak *et al.* 2020) and *Caesalpinia sappan* L. (Kim *et al.* 2021) exist. IQ is a sesquiterpene quinone isolated from the marine sponge *Smenospongia cerebriformis* and inhibits PDK1 activity in cancer cells (Kwak *et al.* 2020). Moreover, *C. sappan* is an herbal medicinal product used to treat algomenorrhea and amenorrhea (Kim *et al.* 2021). Furthermore, *C. sappan* suppresses PDK1 expression and increases mitochondrial ROS levels, which, in turn, promotes endometrial cell apoptosis (Kim *et al.* 2021).

Another candidate drug is the LDHA inhibitors. However, this drug has never been used to treat endometriosis in preclinical studies. In light of previous reports, it can be speculated that the coinactivation of LDHA and PDK1 functions shifts from aerobic glycolysis to the TCA cycle/OXPHOS, causing ROS overproduction and culminating in cell death. LDHA regulates pyruvate production and thus acts as a link between glycolysis and the TCA cycle/OXPHOS (Miao *et al.* 2013). LDHA is elevated in many cancer types. Inhibition of LDHA activity, either by RNA interference or by pharmacological inhibitors, can block tumor growth and progression* in vitro* and* in vivo* (Oermann *et al.* 2012, Miao *et al.* 2013). Specific LDHA inhibitors include a small-molecule inhibitor 3-dihydroxy-6-methyl-7-(phenylmethyl)-4-propylnaphthalene-1-carboxylic acid (FX11; Le *et al.* 2010), N-hydroxy-2-carboxy-substituted indole compounds (Granchi *et al.* 2011), and epigallocatechin (Wang *et al.* 2013). LDHA siRNA or FX11 can effectively inhibit cancer growth through the shift to OXPHOS and increased intracellular ROS (Arseneault *et al.* 2013). From the aforementioned data, inactivating LDHA is possible to inhibit the endometriotic cell growth possibly through ROS overproduction. While revising this manuscript, an interesting paper was reported. The silencing of the LDHA expression in immortalized cells promotes apoptosis through glycolysis inhibition and mitochondrial function suppression (Zheng *et al.* 2021). Future studies are expected to verify the effectiveness of the combination treatment of DCA and LDHA inhibitors in endometriosis. A therapeutic strategy focusing on the shift from aerobic glycolysis to the TCA cycle/OXPHOS may be a promising nonhormonal therapy for endometriosis.

Endometriotic cells are constantly exposed to iron-derived oxidative stress and hypoxic condition, and the shift from aerobic glycolysis to the TCA cycle/OXPHOS may cause ROS overproduction, leading to cell death. Increases in glucose uptake, glycolytic reserve, and gene expression of glycolytic enzymes (HK2, PFKFB3, PKM2, LDHA, and PDK1) are associated with a compensatory decrease in mitochondrial respiration. Molecules that are directly involved in the reprogramming of mitochondrial metabolism may be therapeutic targets for endometriosis (Kobayashi *et al.* 2021*a*). In particular, the metabolic shift may be an attractive target for nonhormone-based endometriosis treatment. Treatment strategies that utilize metabolic reprogramming are implemented not only in cancer but also in sepsis (Bakalov *et al.* 2020), schizophrenia (Pruett & Meador-Woodruff 2020), autoimmune disease (Kornberg 2020), or mitochondrial disease (Kobayashi *et al.* 2021*a*).

## Summary and conclusions

The currently available treatment options for endometriosis suppress ovarian function, but no cure currently exists. Such treatment is unsuitable for women desiring pregnancy. Therefore, studies on new drugs that do not suppress ovarian function have commenced. Several researchers focused on genes and proteins that may affect metabolic pathways to promote endometriotic cell survival and growth. The metabolism characteristic of endometriosis is significantly affected by estrogen (Kobayashi *et al.* 2021*b*). Moreover, estrogen is involved not only in hormonal action but also in various functions (e.g. mitochondrial biosynthesis and energy metabolism). Estrogen can also affect ATP production, energy conversion, ROS production, and antioxidant defense through the regulation of mitochondrial gene expression. Estrogen downstream target genes (e.g. peroxisome proliferator-activated receptor-gamma coactivator 1α), involved in mitochondrial metabolic biosynthesis, may be potential targets for nonhormonal therapy for endometriosis (Kobayashi *et al.* 2021*b*). Basic and preclinical studies are steadily progressing, although these drugs are still far from clinical application.

Endometriotic cells often reprogram their metabolic pathways to adapt to environmental challenges and facilitate survival. Endometriotic cells are essentially exposed to a hypoxic microenvironment. HIF-1- and TGF-β-mediated upregulation of LDHA and PDK1 expression induced by hypoxia and oxidative stress is an adaptive phenomenon in endometriosis (Qi *et al.* 2014, Young *et al.* 2014, 2016, Marianna *et al.* 2017, Horne *et al.* 2019). The actual balance between glycolysis and the TCA cycle/OXPHOS is regulated by glycolytic predominance (Young *et al.* 2014, Marianna *et al.* 2017, Lee *et al.* 2019, Reckzeh *et al.* 2019, Wang *et al.* 2019). This is supported by measurements showing local elevation of HIF-1, TGF-β, PDK1, LDHA, and lactate as well as the counterclockwise rotation of the TCA cycle (i.e. elevated levels of citrate, α-KG, and succinate; Dutta *et al.* 2012, Marianna *et al.* 2017, Karaer *et al.* 2019; Fig. 2). Metabolic changes in endometriosis shift from the TCA cycle/OXPHOS to aerobic glycolysis and suppress ROS overproduction for its survival.

This phenomenon is similar to the Warburg effect in cancer (Kasvandik *et al.* 2016, Liberti & Locasale 2016, Atas *et al.* 2020). Oxidative stress and hypoxia are largely involved in the development and progression of endometriosis, and functional modifications of these signaling pathways may be a viable target for endometriosis treatment (Kasvandik *et al.* 2016). Negative regulation of the Warburg effect can increase endogenous ROS and then induce endometriotic cell death (Lim *et al.* 2021). Therefore, inhibition of PDK and LDHA may be a new strategy in nonhormonal therapy for endometriosis. Currently, a few small-molecule inhibitors and natural compounds have been reported to inhibit PDK (Anwar *et al.* 2021) or LDHA (Wang *et al.* 2013) with promising oral administration (Table 1).

In conclusion, metabolic flexibility in endometriosis is the ability to adapt to environmental changes. The reverse Warburg effect could be an attractive target for developing nonhormonal treatments for endometriosis.

## Declaration of interest

The authors declare that there is no conflict of interest that could be perceived as prejudicing the impartiality of the research reported.

## Funding

This work was supported by the Japan Society for the Promotion of Science (JSPS), grant numbers JP16K11150, 18K09269, and 18K09234.

## Author contribution statement

H K performed the literature search and collected data using the Web database. H K made a contribution to the conception of the study and also contributed to the interpretation of included research studies. The final version of the manuscript has been read and approved by H K.
